# Metabolic Plasticity and Abiotic Stress Adaptation in Freshwater Algae During Phycoremediation of Polluted River Water

**DOI:** 10.1002/pei3.70093

**Published:** 2025-10-18

**Authors:** Dharmendra Kumar, Shivankar Agrawal, Sanjukta Sahoo, Elangbam Geetanjali, Dinabandhu Sahoo

**Affiliations:** ^1^ Department of Botany Miranda House, University of Delhi Delhi India; ^2^ APC Microbiome Ireland, School of Microbiology University College Cork Cork Ireland; ^3^ KIIT Bhubaneshwar Odisha India; ^4^ Department of Botany University of Delhi Delhi India

**Keywords:** biochemical changes, blue‐green bioeconomy, contaminants, sustainable consumption, wastewater valorization

## Abstract

Freshwater algae possess remarkable metabolic flexibility and environmental resilience, enabling them to adapt to polluted habitats and contribute to ecological restoration. This study investigates the physiological and biochemical responses of five green algal taxa: *Monoraphidium* sp., *Scenedesmus* sp., *Nephrocytium* sp., *Chlorococcum* sp., and *Klebsormidium* sp. during a 25‐day phycoremediation of contaminated water of the Yamuna River, New Delhi, India. The water, characterized by high concentrations of organic matter, nutrients, and heavy metals, induced species‐specific metabolic adjustments. A decline in chlorophyll *a* and *b* (31.25% ± 2.25% to 67.11% ± 5.37% and 11.49% ± 0.25% to 86.98% ± 3.21%, respectively) indicated stress or damage to the photosynthetic system. This decline can be caused by various abiotic or biotic stress factors, while carotenoid accumulation, particularly in *Chlorococcum* sp. (307.70% ± 4.32%), suggested photoprotective adaptations. Enhanced biosynthesis of phenolic compounds and flavonoids in *Chlorococcum* sp. (139.33% ± 4.32% and 81.81% ± 2.72%, respectively) correlated with elevated antioxidant activity across all species (27.67% ± 1.61% to 73.51% ± 2.44% DPPH inhibition). Lipid content shifts were species‐dependent, with *Monoraphidium* sp. showing the highest increase (63.02% ± 2.09%). Elemental CHNS analysis revealed increased carbon content and reduced nitrogen and sulfur levels, indicating altered nutrient dynamics. Principal Component Analysis (PCA) elucidated distinct clusters reflecting interspecific differences in stress‐responsive metabolic traits. This study demonstrates the metabolic plasticity and stress tolerance of green algae under complex pollutant loads, advancing our understanding of algal adaptation mechanisms. It shows that phycoremediation not only enhances interspecific biochemical divergence but also alters algal elemental stoichiometry. By integrating multivariate biochemical analysis with CHNS profiling, we identify nitrogen as the primary driver of post‐treatment differentiation. These findings highlight both the ecological and biotechnological relevance of algae in integrated water treatment and sustainable biomass utilization, while offering a novel framework for selecting candidate species in environmental remediation and biotechnological applications.

**Abbreviations**
BBM: BBM media biomass
TB: Treated Biomass after Phycoremediation

## 1 Introduction

Pollutants from human activities, such as agricultural runoff, industrial discharge, and domestic waste, pose significant threats to ecosystems and human health. Even at low concentrations, these contaminants persist, bioaccumulate, and destabilize aquatic ecosystems (Chess et al. 2005). Phycoremediation, a green technology utilizing plants to remove or degrade pollutants, has emerged as a promising solution. However, it faces challenges, including long remediation times, potential ecological disturbances, and limited effectiveness at high pollutant concentrations. Phycoremediation relies on mechanisms such as phytodegradation, phytoextraction, phytovolatilization, and phytostabilization, each targeting specific pollutants (Pivetz 2001). Terrestrial plants, widely studied for their ability to absorb and sequester heavy metals or degrade organic pollutants, require extensive land areas and may disrupt soil ecosystems (Mahar et al. 2016; Saleem et al. 2020). These constraints limit their scalability and efficiency in diverse environments. In contrast, algae present unique advantages for aquatic remediation, including rapid growth, high surface‐to‐volume ratios, and adaptability to a wide range of environmental conditions (Seymour et al. 2017; Subashchandrabose et al. 2011).

Algae remove pollutants from contaminated water bodies through the mechanism of phycoremediation. This process involves the absorption, accumulation, and detoxification of pollutants by algae, which play a crucial role in improving water quality. Algae use their metabolic pathways to absorb various contaminants, such as organic compounds, nitrates, phosphates, heavy metals, and other harmful substances, thus effectively cleaning the water (Upadhyay et al. 2018; Koul and Taak 2018). Additionally, algae can enhance the degradation of these pollutants by metabolizing them into less harmful substances, making them an efficient and sustainable solution for environmental remediation. Despite these benefits, challenges such as maintaining algal cultures in polluted environments, controlling biofouling, and addressing the ecological risks of introducing non‐native species must be considered when implementing algae‐based remediation systems.

Algae's metabolic flexibility enables efficient absorption of nutrients and contaminants, even in nutrient‐poor or heavily polluted water bodies. Additionally, algae can function in extreme environments, such as those with high salinity, extreme pH, or elevated pollutant concentrations, making them promising candidates for large‐scale aquatic phycoremediation. However, their remediation ability depends on factors like light and pollutant toxicity, which affect their growth and function. Under stress, algae adjust by producing metabolites such as antioxidants, osmolytes, and lipids that help them stay resilient. These responses not only aid in detoxification but also result in metabolite‐enriched biomass with potential high‐value applications (Kumar et al. 2014; Cao et al. 2017; Rugiu et al. 2021). Despite extensive research on algae's pollutant removal capabilities, microalgal biomass obtained from wastewater phycoremediation provides proteins, lipids, antioxidants, and bioproducts (Apandi et al. 2019) and is used as food for marine species, ultimately reducing both wastewater treatment and mariculture costs (de Alva and Pabello 2021). Limited attention has been paid to the metabolic changes that occur during and after phycoremediation, as well as to the enriched algal biomass for biofuels, nutraceuticals, or pharmaceuticals. Exploring these post‐remediation biochemical dynamics can enhance the dual benefits of phycoremediation: environmental detoxification and the sustainable use of biomass.

Apart from their effectiveness in pollutant removal, *Monoraphidium* sp., *Scenedesmus* sp., *Nephrocytium* sp., *Chlorococcum* sp., and *Klebsormidium* sp. are highly valued for commercial applications because they produce substantial amounts of metabolites useful for biofuel and pharmaceutical industries. *Scenedesmus* sp. and *Chlorococcum* sp. are especially notable for their high lipid content and fast growth, making them suitable for biodiesel production, while *Monoraphidium* sp. excels at accumulating polyunsaturated fatty acids important for nutraceuticals and energy products. *Klebsormidium* sp. is distinguished by its tolerance to stress and its ability to generate significant fatty acids, antioxidants, and anti‐inflammatory compounds, and *Nephrocytium* sp. is valued for its carotenoid production, which is applicable in pharmaceuticals and cosmetics. While *Scenedesmus* sp. and *Chlorococcum* sp. are preferred for large‐scale biofuel efforts, the combined metabolite profiles of all five species make them attractive sources for a wide range of commercial products beyond environmental remediation (Singh et al. 2023; Segura‐Morales et al. 2024). This study investigates the biochemical responses of five algal species: *Monoraphidium* sp., *Scenedesmus* sp., *Nephrocytium* sp., *Chlorococcum* sp., and *Klebsormidium* sp., following their use in the phycoremediation of polluted water from the Yamuna River. Changes in chlorophyll, carotenoids, phenolic compounds, flavonoids, and lipid content were observed, reflecting the algae's metabolic adjustments to environmental stressors such as heavy metals and organic pollutants. These stressors induce oxidative stress, prompting the algae to activate detoxification pathways. The study highlights species‐specific adaptations to different contaminants, providing insights into the mechanisms underlying pollutant removal. The selected algae were chosen for their metabolic diversity and resilience, making them promising candidates not only for effective phycoremediation but also for sustainable applications in biofuel production and pharmaceuticals. Overall, the findings support the dual potential of these algae in environmental cleanup and biotechnological innovation.

## 2 Material and Methods

Algal biomass obtained from the phycoremediation of Yamuna water with biomass grown in Bold's Basal Medium (BBM) as a control. Five algal species *Monoraphidium* sp., *Scenedesmus* sp., *Nephrocytium* sp., *Chlorococcum* sp., and *Klebsormidium* sp. were chosen for their adaptability, rapid growth, and metabolic diversity. The algae *Monoraphidium* sp., *Scenedesmus* sp., *Nephrocytium* sp., *Chlorococcum* sp., and *Klebsormidium* sp. all belong to the domain Eukaryota and the kingdom Plantae but differ in their lower taxonomic ranks and evolutionary lineages. *Monoraphidium* and *Scenedesmus* belong to the division Chlorophyta, class Chlorophyceae, and order *Sphaeropleales* but differ at the family level; *Monoraphidium* is in Selenastraceae, while *Scenedesmus* is in Scenedesmaceae. *Nephrocytium* also falls under the same division, class, and order but belongs to the family Neochloridaceae. *Chlorococcum* shares the same division and class but is classified under the order Chlorococcales and family Chlorococcaceae. In contrast, *Klebsormidium* is taxonomically and evolutionarily distinct, belonging to the division Charophyta (sometimes referred to as Streptophyta), class Klebsormidiophyceae, order Klebsormidiales, and family Klebsormidiaceae. These species are ideal for studying pollutant‐induced metabolic changes due to their high tolerance to pollutants and antioxidant production. These algae were used for the removal of pollutants (nitrate, phosphate, chloride, sulphate, silicon, ammonia, and heavy metals). All these pollutants were found in very high concentrations in Yamuna water, far exceeding the WHO‐prescribed limits, as reported by Kumar et al. (2023a, 2023b) and Kumar and Sahoo (2024).

### 2.1 Isolation of Inhabitant Algal Species

Water samples were collected from the heavily polluted Yamuna River near the Signature Bridge in Wazirabad, Delhi, India (28.705643° N, 77.233436° E). This site is highly contaminated due to industrial effluents, sewage, and agricultural runoff, making it an ideal case study for evaluating algal efficacy in extreme conditions. However, the high pollutant load may hinder the growth of some algal species, limiting certain remediation approaches.

Sampling was conducted in sterilized, air‐tight bottles to prevent contamination, and the collected water was immediately stored at 4°C until further analysis. Indigenous algae were isolated using standard microbiological techniques, including streaking on agar plates, serial dilution, and subsequent culture in BBM. Growth conditions were maintained at a pH of 7.2, a temperature of 26°C, and a light intensity of 6000 lx. Algal species were identified morphologically using standard references (Bellinger and Sigee 2015; Sheath and Wehr 2015; Guiry 2013; Sahoo and Seckbach 2015) and examined under a Nikon Eclipse TE2000‐U microscope. The algal species were isolated and identified, and the culture medium used for their growth was Bold's Basal Medium (BBM), which served as the control. These algal species were then utilized for phycoremediation, and the biochemical changes that occurred during the process were studied. All measurements were performed in triplicate, and absorption was measured using the Shimadzu UV–VIS 1900 spectrophotometer.

### 2.2 Phycoremediation

Phycoremediation experiments were performed in 2 L glass flasks containing Yamuna water samples, mixed with a 10% (v/v) stock culture of each isolated alga. Initial optical density was standardized to 2.0 at 680 nm to ensure consistent starting conditions. Cultures were maintained under controlled conditions (26°C, light intensity of 6000 lx, and a 16:8‐h light–dark photoperiod) for 25 days. Following remediation, algal biomass was harvested by centrifugation at 5000 rpm, washed thoroughly to remove residual pollutants, and lyophilized for biochemical analyses.

### 2.3 Preparation of Algal Extract for Phenol, Flavonoids, and DPPH Analysis

1 g of lyophilized algal material was first homogenized with 5 mL of 95% (v/v) methanol in a test tube. This initial mixture was then placed in a shaker at room temperature and agitated for 30 min to ensure thorough mixing and effective extraction of the algal compounds. This process was repeated three times for complete extraction, with each cycle using fresh methanol to further extract any remaining compounds from the algal biomass. After completing these three extraction cycles, the combined methanol extracts were pooled together. The pooled methanol solution was then subjected to evaporation in an oven set at 60°C to remove all the solvent. This evaporation step continued until only the dried extract remained. The resultant dried extract was carefully collected and stored properly for subsequent analysis.

### 2.4 Estimation of Chlorophyll Pigments

The extraction and determination of chlorophyll pigments were performed as per the protocol of Yang et al. (1998) with slight modifications. 1 g of the powdered algal sample was homogenized with 10 mL of 100% methanol in a glass tube for 30 min in an incubator shaker at room temperature in the dark. After incubation, the tube was centrifuged at 5000 rpm to separate the supernatant. To ensure complete extraction of chlorophyll pigments, the process was repeated three times, and each extract was collected into separate tubes. The combined extracts were then used for the determination of chlorophyll concentrations. All readings were recorded in the dark to prevent degradation of the chlorophyll pigments, using methanol as the blank. The absorbance of the extracts was measured at specific wavelengths (663.6 nm for Chl a, 646.6 nm for Chl b, and 470.0 nm for carotenoids). The concentrations of chlorophyll a, chlorophyll b, and carotenoids were calculated using the following equations:

$$\text{Chlorophyll}\;a\;\left(\mathrm{\mu g}/g\right):12.25A_{663.6}–2.25A_{646.6}$$

$$\text{Chlorophyll}\;b\;\left(\mathrm{\mu g}/g\right):20.{\text{9A}}_{646.6}–4.91A_{663.6}$$

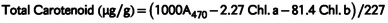




### 2.5 Estimation of Total Lipid

Total lipid content in algae was determined according to the protocol of Folch et al. (1957). 1 g of lyophilized algal biomass was mixed with 5 mL of a chloroform‐methanol mixture (2:1, v/v) and placed in an incubator shaker for 1 h at room temperature to ensure thorough extraction of lipids. After incubation, the mixture was thoroughly mixed, which caused it to separate into two distinct layers: the upper layer containing the lipids and the lower layer with other components. The extraction process was repeated three times to ensure maximum lipid recovery. For each extraction, the upper lipid‐containing layer was carefully collected and transferred into separate tubes. After all three extractions, the combined lipid layers were evaporated using nitrogen gas to remove the solvent, and total lipid content was then calculated using the following formula:

$$\%\text{Total lipid}=\frac{\left(W0-W1\right)}{\text{Weight of biomass}\;\left(g\right)}\times 100$$

where W0, Initial weight of tube and W1, Final weight of tube.

### 2.6 Estimation of Total Phenol Content

The total phenolic content (TPC) was estimated using the Folin–Ciocalteu colorimetric method, as described by Singleton and Rossi (1965), with slight modifications. To begin, 100 μL of the algal extract was carefully pipetted into a clean test tube. To this, 400 μL of Folin–Ciocalteu's phenol reagent, previously diluted tenfold with deionized water, was added. The mixture was vortexed vigorously for 2 min to ensure thorough interaction between the extract and the reagent. After vortexing, the mixture was allowed to stand undisturbed for 5 min at room temperature to facilitate the initial reaction. Subsequently, 500 μL of a freshly prepared 7.5% (w/v) sodium bicarbonate solution was added to the mixture. The reaction mixture was then incubated for 90 min in the dark at room temperature to prevent any photo‐degradation of phenolic compounds. After the incubation period, the absorbance of the reaction mixture was measured at a wavelength of 760 nm using a Shimadzu UV‐1900 spectrophotometer. Deionized water was used as the blank for calibration. The TPC in the algal extract was determined by comparing the absorbance values to a standard curve prepared using gallic acid solutions of known concentrations. The standard curve was generated by serially diluting gallic acid to obtain concentrations ranging from 10 – 400 mg/L. The results were expressed as milligrams of gallic acid equivalents per gram of dry weight of the sample (mg GAE/g DW), indicating the phenolic content present in the algal biomass.

### 2.7 Estimation of Total Flavonoid Content

Total flavonoid content was determined using the aluminum chloride colorimetric method as described by (Ribarova et al. 2005; Miliauskas et al. 2004). Specifically, 0.6 mL of the prepared microalgal extract was transferred to a clean test tube, followed by the addition of 0.6 mL of freshly prepared 2% aluminum chloride (AlCl_3_) solution. The mixture was thoroughly vortexed to ensure homogeneity and incubated for precisely 60 min at room temperature to allow the formation of the flavonoid‐AlCl_3_ complex. After incubation, the absorbance of the mixture was measured at 420 nm using a Shimadzu UV‐1900 spectrophotometer, with methanol serving as the blank. A standard calibration curve was prepared using quercetin as the reference compound: a stock solution was made by dissolving 5 mg of quercetin in 1.0 mL of methanol, followed by serial dilutions to prepare standard solutions with concentrations ranging from 5 – 200 μg/mL. The absorbance of these standards was measured under identical conditions, and a standard curve was plotted. The total flavonoid content in the algal samples was calculated by interpolating the absorbance values onto the standard curve and expressed as milligrams of quercetin equivalents per gram of dry weight of the algal biomass (mg QE/g DW).

### 2.8 Estimation of 2,2‐Diphenyl‐1‐Picrylhydrazyl Free Radical Scavenging Assay (DPPH)

The antioxidant activity of the algal extracts was determined using the DPPH (2,2‐diphenyl‐1‐picrylhydrazyl) radical scavenging assay, following the protocol described by Cheng et al. (2006) with slight modifications to ensure reproducibility. A stock solution of DPPH (0.1 mM) was prepared freshly in methanol and stored in an amber bottle to prevent degradation by light. For the assay, 200 μL of the DPPH solution was mixed thoroughly with 200 μL of the algal extract in a 96‐well microplate or test tube. The mixture was incubated in the dark at a controlled temperature of 37°C for exactly 1 h to ensure consistency and prevent light‐induced reactions that could interfere with the assay. After the incubation period, the absorbance of the reaction mixture was measured at 515 nm using a Shimadzu UV‐1900 spectrophotometer, with methanol serving as the blank. Gallic acid was used as the positive control, and its reaction was prepared under the same conditions for calibration. The percentage of DPPH radical scavenging activity was calculated using the formula:

$$\text{DPPH scavenging activity}\;\left(\%\right)=\left\{1-\frac{\left(\text{sample}-\text{blank}\right)}{\left(\text{control}-\text{blank}\right)}\right\}\times 100$$

where the sample absorbance represents the absorbance of the algal extract with DPPH, the blank absorbance is that of methanol without DPPH, and the control absorbance is that of DPPH solution alone without the algal extract.

### 2.9 Estimation of Carbon, Hydrogen, Nitrogen, and Sulfur (CHNS)

CHNS composition of dried algal biomass was estimated using an Elementar analysis system (Elementar Analysensysteme GmbH, Germany, Vario EL V 3.000).

### 2.10 Statistical Analysis

All analyses were conducted in triplicate, and the results were expressed as mean ± standard deviation (SD). The significance of differences among metabolites was determined using Analysis of Variance (ANOVA) in Excel, with a significance threshold set at *p* < 0.05. Principal Component Analysis (PCA) and Agglomerative Hierarchical Clustering (AHC) were employed for a detailed statistical analysis of the biochemical dataset. PCA and AHC were performed using Origin 9.0.

## 3 Results

Growth rate analysis of these algae demonstrates that, under BBM (Bold's Basal Medium) and controlled experimental conditions, all species exhibited a similar growth trajectory, actively expanding until reaching a plateau around 20 days, then surviving in a stationary phase for an additional 15 days; however, when cultivated in Yamuna water, variations in growth rates emerged among the species. This observation indicates that metabolite yield differences in standardized laboratory medium result primarily from physiological adaptation rather than culture phase or timing. In contrast, growth variations in Yamuna water reflect species‐specific adaptive strategies and stress tolerance. The dominance of these genera in the polluted Yamuna River highlights their metabolic and physiological flexibility, allowing them to remain viable and metabolically active despite halted cellular proliferation.

### 3.1 Biochemical Shifts in Algae After Phycoremediation Treatment

The percentage changes in various biochemical compounds across five algal species were shown in Figure 6. These findings highlight varying responses to stress and pollutant exposure across different algal species, with some showing enhanced antioxidant activity and lipid production, which could be useful for biotechnological applications.

### 3.2 Phycoremediation Analysis

All the algae used in the phycoremediation of polluted Yamuna water effectively remove pollutants, with nitrate and phosphate levels reduced by up to 99%, and heavy metals decreased by as much as 85%, along with other types of contaminants, as reported by Kumar et al. (2023a, 2023b) and Kumar and Sahoo (2024).

### 3.3 Pigment Analysis

**FIGURE 1:**
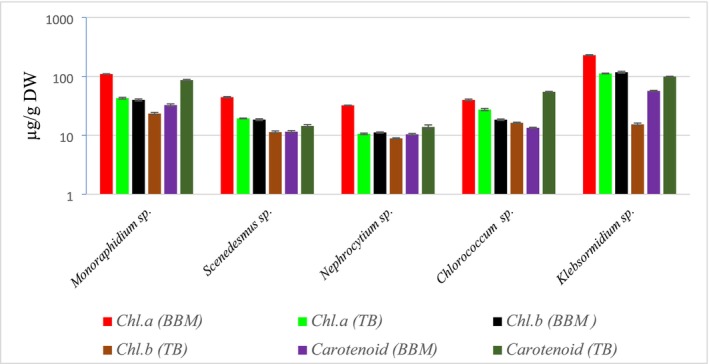
Chl. a, Chl. b, and Carotenoid content in Bold's Basal Medium (BBM) and Treated Biomass (TB) of algal species.

Pigment analysis of algae, as shown in Figure 1, reveals notable differences in chlorophyll and carotenoid content across species and culture media (BBM and TB). *Klebsormidium* sp. exhibited the highest levels of both chlorophyll a and b, especially in BBM medium, with Chl. a reaching 229.16 μg/g DW and Chl. b at 117.7 μg/g DW. In contrast, *Nephrocytium* sp. showed the lowest pigment concentrations, particularly in TB medium (e.g., 10.60 μg/g DW for Chl. a and 8.86 μg/g DW for Chl. b). *Monoraphidium* sp. displayed moderate Chl. a levels but stood out for its carotenoid content, recording 86.51 μg/g DW in TB medium, surpassed only by *Klebsormidium* sp. with 99.24 μg/g DW. Generally, pigment concentrations were higher in BBM than in TB for most species, except for carotenoids, which were often elevated in TB.

### 3.4 Phenol

**FIGURE 2:**
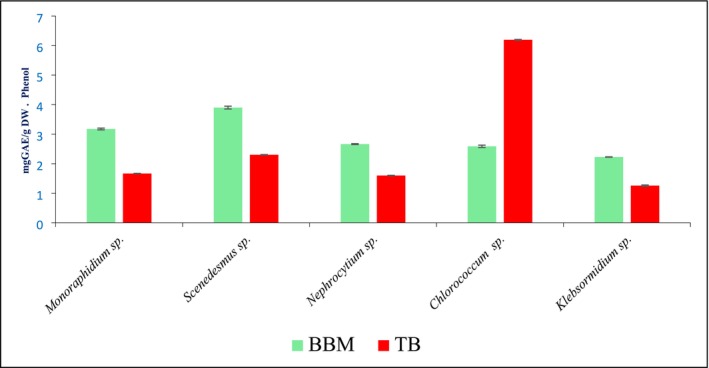
Phenol content in BBM and TB of algal species (* shows increase in concentration after phycoremediation).

The phenolic content, expressed as mg GAE/g DW, varied notably among the five microalgal species grown in BBM and TB media (Figure 2). In general, higher phenolic levels were observed in BBM compared to TB for most species, with the exception of *Chlorococcum* sp., which exhibited a significantly higher phenolic content in TB (6.19 ± 0.11) than in BBM (2.58 ± 0.03). *Scenedesmus* sp. showed the highest phenolic concentration in BBM (3.89 ± 0.04), followed by *Monoraphidium* sp. (3.17 ± 0.029). In contrast, *Klebsormidium* sp. and *Nephrocytium* sp. exhibited relatively lower phenolic levels in both media, with *Klebsormidium* sp. showing the lowest value in TB (1.25 ± 0.021). These results indicate that both species and growth medium significantly influence phenolic production.

### 3.5 Flavonoids

**FIGURE 3:**
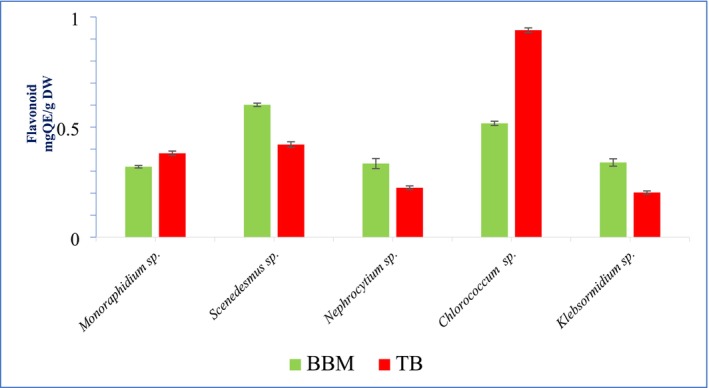
Flavonoid content in BBM and TB of algal species.

The flavonoid content, expressed in mg quercetin equivalents per gram dry weight (mg QE/g DW), varied among microalgal species and cultivation media (BBM and TB). In general, *Scenedesmus* sp. exhibited the highest flavonoid levels in BBM (0.601 ± 0.07), while *Chlorococcum* sp. had the highest in TB (0.94 ± 0.0105). *Monoraphidium* sp. showed slightly higher flavonoid content in TB (0.38 ± 0.010) compared to BBM (0.32 ± 0.0055), while *Nephrocytium* sp. and *Klebsormidium* sp. had reduced flavonoid levels in TB relative to BBM. These differences highlight the influence of culture media on flavonoid biosynthesis in microalgae (Figure 3).

### 3.6 DPPH Scavenging Activity

**FIGURE 4:**
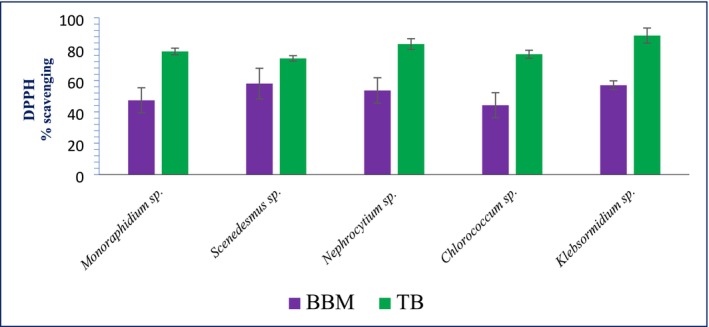
DPPH scavenging activity in BBM and TB of algal species.

The DPPH analysis, presented in Figure 4, reveals a consistent trend across all algal species: higher DPPH scavenging activity (%) was observed when cultured in TB medium compared to BBM. Among the species tested, *Klebsormidium* sp. exhibited the highest antioxidant activity with values of 56.97% ± 2.8% in BBM and 88.67% ± 4.82% in TB. *Nephrocytium* sp. also showed strong activity, increasing from 53.63% ± 2.12% (BBM) to 83.23% ± 3.42% (TB). *Scenedesmus* sp. followed a similar pattern, with values rising from 58.03% ± 4.8% to 74.08% ± 1.75%. *Monoraphidium* sp. demonstrated moderate activity, improving from 47.35% ± 3.02% to 78.53% ± 2.1%, while *Chlorococcum* sp. recorded the lowest values at 44.23% ± 4.0% in BBM and 76.75% ± 2.57% in TB. These results suggest that TB medium enhances the antioxidant potential of microalgal species more effectively than BBM.

### 3.7 Total Lipid

**FIGURE 5:**
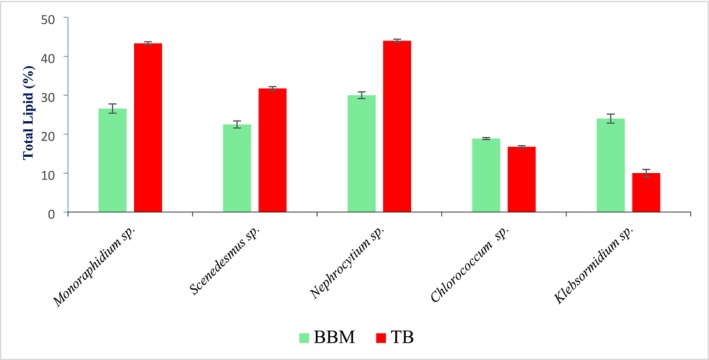
Total lipid content in BBM and TB of algal species.

The analysis of total lipid content revealed distinct differences between biomass cultivated in Bold's Basal Medium (BBM) and that treated with an alternative biomass treatment (TB). Among the microalgal species studied, *Monoraphidium* sp. exhibited a notable increase in lipid content from 26.58% ± 1.2% in BBM to 43.33% ± 1.42% in TB, indicating enhanced lipid accumulation under treated conditions. *Scenedesmus* sp. showed a similar trend, with lipid content rising from 22.5% ± 0.9% (BBM) to 31.75% ± 1.50% (TB). *Nephrocytium* sp. also responded positively to treatment, increasing from 30% ± 0.867% in BBM to 44.0% ± 1.39% in TB. Conversely, *Chlorococcum* sp. showed a slight decrease in lipid content from 18.88% ± 0.256% (BBM) to 16.76% ± 0.29% (TB). The most marked decline was observed in *Klebsormidium* sp., with lipid content dropping significantly from 24% ± 0.167% in BBM to 10.0% ± 0.95% in TB. These variations suggest species‐specific responses to the treatment, with some benefiting substantially while others show reduced lipid accumulation (Figure 5).

### 3.8 Biochemical Shifts in Algae After Phycoremediation Treatment

**FIGURE 6:**
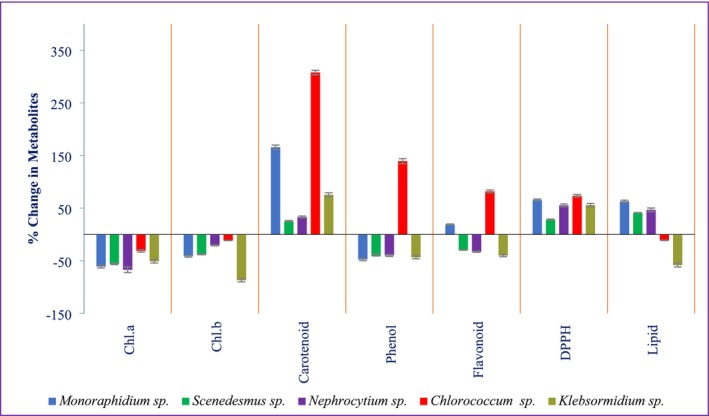
Metabolic change in algal species after phycoremediation.

The percentage changes in various biochemical compounds across five algal species were shown in Figure 6. Among the algae analyzed, *Chlorococcum* sp. exhibited the most pronounced biochemical enhancements, with carotenoids increasing by 307.69% ± 4.32% and phenolic content by 139.33% ± 4.69%, indicating a strong antioxidant response. In contrast, pigment degradation was most severe in *Nephrocytium* sp., which showed the largest reduction in chlorophyll a (−67.11% ± 5.37%), while *Klebsormidium* sp. experienced the greatest decline in chlorophyll b (−86.97% ± 3.21%) and flavonoids (−40.18% ± 2.13%), suggesting high sensitivity to the stress conditions. For free radical scavenging activity, *Chlorococcum* sp. again stood out, demonstrating the highest increase in DPPH activity (73.51% ± 2.44%). Lipid accumulation varied among species, with *Monoraphidium* sp. showing the highest increase (63.01% ± 2.08%), whereas *Klebsormidium* sp. again showed the most substantial decline (−58.33% ± 3.49%). These differential biochemical responses highlight the species‐specific adaptability of algae, with potential implications for their use in biotechnology, particularly in stress resilience and antioxidant production.

### 3.9 CHNS Analysis

**FIGURE 7:**
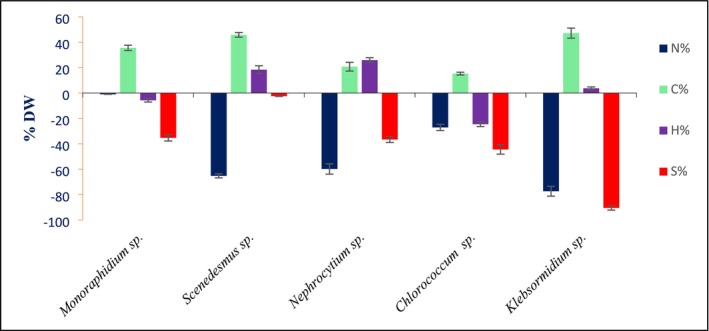
Change in CHNS content in algal species after phycoremedtion.

The CHNS analysis of both BBM and treated biomass, illustrated in Figure 7, shows notable compositional shifts across the different algae species studied. Nitrogen content experiences a marked decline in all species, with the most substantial reduction observed in *Klebsormidium* sp. (around 77%) and a minimal decrease in *Monoraphidium* sp. (less than 1%). Conversely, carbon content rises in all algae, most prominently in *Klebsormidium* sp., which shows nearly a 47% increase, while *Chlorococcum* sp. exhibits the smallest gain at approximately 15%. Sulfur levels drop universally, with *Klebsormidium* sp. again showing the largest decrease (~90%), whereas *Scenedesmus* sp. experiences a relatively minor reduction (~2%). Hydrogen content varies more between species, increasing significantly in *Nephrocytium* sp. by about 26%, but decreasing in *Chlorococcum* sp. by nearly 25%.

### 3.10 Statistical Analysis

A one‐way ANOVA analysis shows that the data for metabolites in the algal sample is highly significant, with *p*‐values of 1.10523E‐25 (*p* < 0.05) and 4.70447E‐25 in CHNS analysis. These values indicate a very low probability of the results occurring by chance. Additionally, the *F* value of 83.64989286 exceeds the critical *F* value of 1.961218, further supporting the significance of the observed differences. This suggests that the variations in metabolites are statistically significant and not due to random fluctuations (Table 1).

**TABLE 1:** One‐way ANOVA analysis of biochemical compounds and CHNS.

	ANOVA						
	Source of variation	SS	df	MS	*F*	*p* *	*F* crit
Biochemical Compounds	Between Groups	31221.55007	13	2401.657698	83.64989286	1.10523E‐25	1.9612184
CHNS	Between Groups	19293.12594	5	3858.625188	91.0287349	4.70447E‐25	2.386069

* The *p*‐values for Biochemical compounds and CHNS are 1.10523E‐25 and 4.70447E‐25. Since these *p*‐values are less than 0.05, the data are considered statistically significant.

### 3.11 PCA Analysis

**FIGURE 8:**
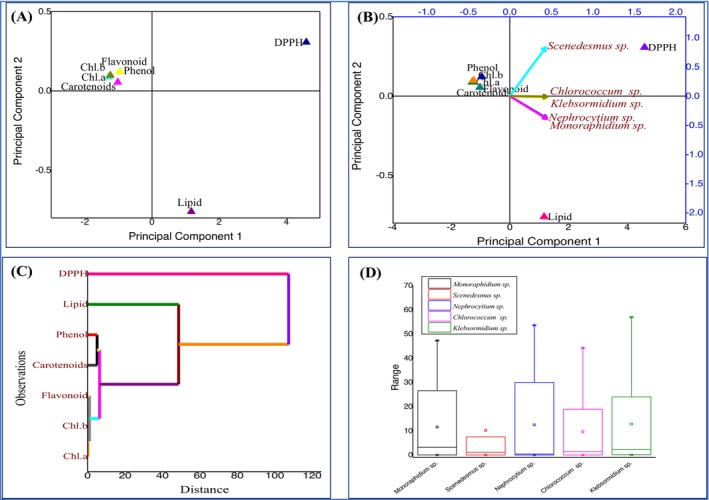
PCA analysis of algae before phycoremediation (A) Score plot, (B) Biplot, (C) AHC, (D) Boxplot.

**FIGURE 9:**
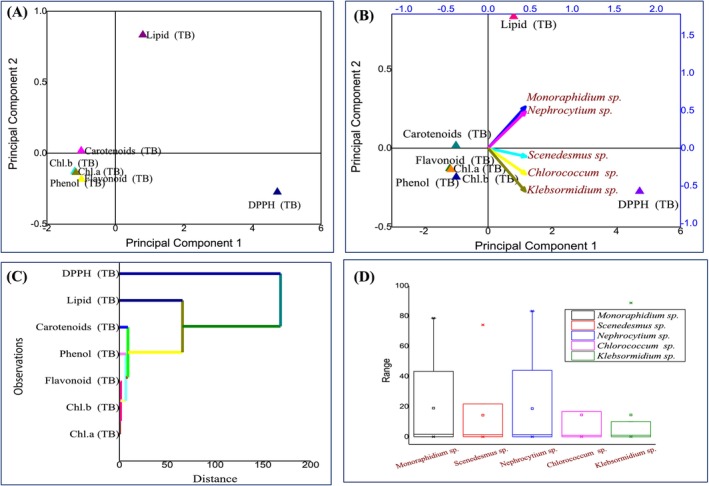
PCA analysis of algae after phycoremediation (A) Score plot, (B) Biplot, (C) AHC, (D) Boxplot.

**FIGURE 10:**
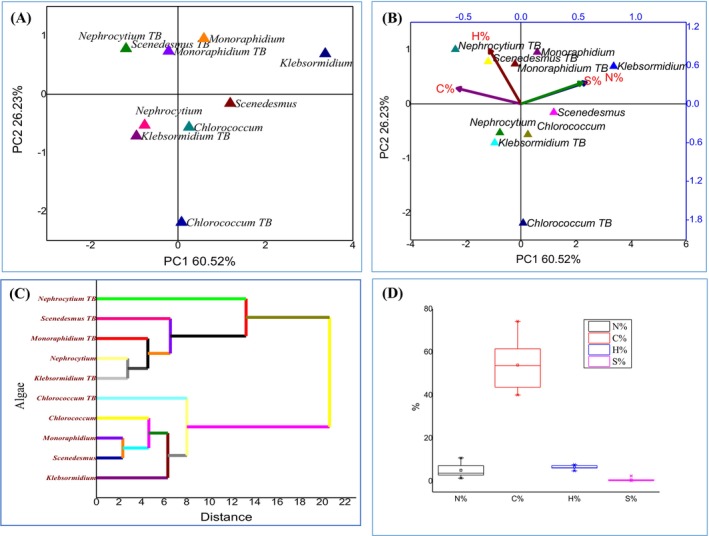
PCA analysis of CHNS content in algae before and after phycoremediation (A) Score plot, (B) Biplot, (C) AHC, (D) Boxplot.

The hypothesis tested in this study was that different algal species exhibit distinct biochemical profiles before and after phycoremediation, which can be identified using multivariate statistical approaches. PCA was chosen because it effectively reduces dimensionality while retaining variation, thereby allowing the identification of key traits driving species separation. PCA and complementary multivariate analyses were performed to assess the biochemical and elemental responses of algae before and after phycoremediation (Figures 8, 9, 10). Prior to treatment (Figure 8), *Scenedesmus* sp. appeared as the most biochemically distinct species, primarily influenced by lipids and antioxidant activity (DPPH), while *Monoraphidium* sp. showed the least variation; hierarchical clustering confirmed functional groupings of biochemical traits (lipids with DPPH, pigments with secondary metabolites), and boxplots indicated greater variability in *Scenedesmus* sp. and *Klebsormidium* sp. After phycoremediation (Figure 9), species separation became more pronounced, with *Monoraphidium* sp. and *Nephrocytium* sp. aligning with the first principal component and showing the highest variability, whereas *Chloreococcum* sp. and *Klebsormidium* sp. remained more stable, as reflected in the score plot, biplot, clustering, and boxplot analyses. Analysis of CHNS content (Figure 10) further revealed distinct grouping of pre‐ and post‐treatment samples, with nitrogen (N%) and sulfur (S%) driving separation along one axis and carbon (C%) and hydrogen (H%) along another; nitrogen emerged as the most variable and responsive element, while sulfur and hydrogen remained relatively stable. Collectively, these results demonstrate that phycoremediation enhances interspecific biochemical divergence and induces significant shifts in algal elemental stoichiometry, with nitrogen being the key driver of post‐treatment differentiation.

The results of this study are crucial for validating the observed biochemical changes in algae, ensuring their consistency and reliability. The analysis of biochemical composition, pigments, antioxidants, lipids, and CHNS content in algae grown in BBM media and treated Yamuna water reveals distinct variations in metabolic responses. For instance, *Klebsormidium* sp. shows the highest concentrations of chlorophyll and carotenoids in BBM, while *Nephrocytium* sp. demonstrates the lowest pigment concentrations, indicating species‐specific responses to nutrient availability. Variations in lipid content and DPPH antioxidant activity highlight how algae adapt to environmental stress and pollutant exposure. CHNS analysis further shows shifts in elemental composition, with increased carbon and hydrogen and decreased nitrogen and sulfur, reflecting metabolic adjustments to stress. These findings emphasize the algae's adaptability, which has significant implications for bioremediation and biomass applications.

## 4 Discussion

Testing multiple algal strains for the same metabolites in phycoremediation provides basic comparisons but can be limiting due to their diverse evolutionary adaptations. Chlorophytes such as *Scenedesmus* and *Monoraphidium* produce secondary carotenoids under stress, whereas streptophytic algae like *Klebsormidium* rely on different tolerance mechanisms. Despite algae's known remediation potential, species‐specific biochemical responses remain underexplored. Targeted screening of metabolic traits would improve understanding of adaptation and strain selection.

Algae adapt to stress by modulating phenols, flavonoids, pigments, and lipids, enhancing antioxidant defenses, stabilizing membranes, and balancing light absorption. Indigenous algae thus contribute to river self‐purification, nutrient and metal sequestration, and ecosystem resilience under climate‐driven stress (Hellier et al. 2015). Nutrient limitation often triggers starch, lipid, carotenoid, astaxanthin, PUFA, and TAG accumulation (Gifuni et al. 2019), as also observed after 25 days of phycoremediation in this study. Among species tested, *Chlorococcum* sp. showed the greatest increases in carotenoids (307.69% ± 4.32%) and phenols (139.33% ± 4.69%), while *Nephrocytium* sp. and *Klebsormidium* sp. exhibited the largest chlorophyll declines.

Pigment shifts corroborate previous findings that environmental stress alters chlorophyll and carotenoids (Maltsev et al. 2021; Dai et al. 2016). Declines in Chl. a under Yamuna water stress align with reports linking ROS‐induced damage and enzymatic degradation (Christ and Hörtensteiner 2014; Nowicka 2022). Similar Chl. a reductions were documented under nitrogen starvation (Al‐Rashed et al. 2016) and cadmium stress (Cheng et al. 2016). Chl. b also declined across species, consistent with metal and nutrient stress studies (Wong et al. 1994; Upadhyay et al. 2022). However, some contrasts exist: *Chlorella vulgaris* showed Chl. b increases under drought or nitrogen stress (Kusvuran 2021; Farooq et al. 2022), suggesting species‐specific roles in adaptation.

Carotenoid accumulation, especially in *Chlorococcum* and *Monoraphidium*, highlights their protective role against oxidative stress (Boussiba 2000; Sánchez et al. 2008). Similar responses were reported in *Dunaliella* (Lin et al. 2017; Wu et al. 2018), *Chlorella* (Rodrigues et al. 2014; Wang et al. 2015), and *Arthrospira* (Sukumaran et al. 2014). Stress‐driven increases (Upadhyay et al. 2022) underscore carotenoids' function in mitigating pollutant‐induced ROS (Pinto et al. 2003; Bhattacharya and Pal 2011).

Phenolic and flavonoid changes were species‐dependent. *Chlorococcum* sp. showed notable increases, consistent with Mukherjee et al. (2020), whereas *Chlorella* and *Phaeodactylum* sometimes decrease phenolics under nutrient stress (Goiris et al. 2015). Flavonoid increases in *Chlorococcum* and *Monoraphidium* align with Mukherjee et al. (2020). These dynamic responses underscore their role in oxidative stress adaptation, though future work should validate compounds via HPLC/LC–MS.

High DPPH scavenging activity in *Monoraphidium* and *Chlorococcum* reflects stress‐induced antioxidant production (Zandi and Schnug 2022), consistent with *Dunaliella* and *Scenedesmus* responses under salinity and Pb(II) exposure (Singh et al. 2016; Danouche et al. 2020). This aligns with reports of enhanced antioxidant activity under wastewater treatment and nutrient stress (Ugya et al. 2021).

Lipid responses varied: *Monoraphidium*, *Scenedesmus*, and *Nephrocytium* increased lipid accumulation, consistent with nitrogen depletion studies (Hsieh and Wu 2009; Chokshi et al. 2017; Yang et al. 2018). In contrast, *Chlorococcum* and *Klebsormidium* showed decreases, diverging from typical trends (Narayanan et al. 2025). Heavy metals and nutrients modulate algal metabolism by acting either as essential cofactors or as stress‐inducing agents, depending on their concentrations. At low levels, metals such as copper and iron are required for key processes, including photosynthesis and nitrogen assimilation, whereas higher concentrations of toxic metals such as cadmium and lead induce oxidative stress, activate antioxidant defenses, and promote chelator synthesis. Similarly, nutrients like nitrogen and phosphate directly regulate growth and lipid metabolism, while nutrient limitation diverts carbon flux toward storage lipids and protective secondary metabolites. These responses underscore the metabolic plasticity of algae in adapting to environmental stressors (Nowicka 2022). Such reductions may reflect carbon flux shifts toward carbohydrates or extracellular polymeric substances (EPS) (Shekh et al. 2016), highlighting species‐specific metabolic priorities.

CHNS analysis confirmed nitrogen and sulfur depletion with increased carbon content, reflecting nutrient uptake and stress adaptation (Renuka et al. 2015). Hydrogen shifts indicated altered storage compound accumulation. Similar changes under sulfur or nitrogen stress were reported in *Dunaliella* and other algae (Skjånes et al. 2013; Singh et al. 2024).

These biochemical and elemental shifts have biotechnological implications. Carbon flux toward lipids and polymers under nutrient stress supports biofuel and bioplastic production (Iqbal et al. 2025), while enriched proteins and fatty acids in biomass make algae promising for aquafeeds. Metal sequestration and nutrient recycling enhance their use as biofertilizers (Nagappan et al. 2021).

Metabolic shifts in algae driven by heavy metals and nutrient availability hold promise for sustainable biotechnologies. Under nutrient stress, algae redirect carbon into biopolymers and lipids, serving as precursors for bioplastics and offering scalable, carbon‐capturing alternatives to fossil plastics; engineered strains further enhance yield and quality (Iqbal et al. 2025). Their ability to accumulate proteins, fatty acids, and micronutrients enables direct use in aquafeed, reducing reliance on fishmeal and fish oil, improving feed efficiency, and lowering ecological impacts. Moreover, metal‐induced responses allow immobilization and transformation of contaminants, while nutrient recycling enriches biomass with bioactive compounds, making algal products valuable biofertilizers that enhance soil fertility, aid remediation, and reduce chemical inputs (Nagappan et al. 2021). This study reveals key biochemical and elemental responses during phycoremediation but has limitations: lab‐cultured isolates may not reflect in situ dynamics, molecular barcoding was absent, and pollution variability complicates interpretation. Future research should integrate multi‐season field trials, molecular tools, and continuous pollution monitoring. Limitations include reliance on lab‐cultured isolates rather than in situ communities, lack of molecular barcoding, and absence of continuous pollution monitoring. Future work should integrate multi‐season trials, molecular identification, and standardized contaminant profiling for greater ecological realism.

### 4.1 Principal Component Analysis (PCA), Agglomerative Hierarchical Clustering (AHC), and BOX Plot Analysis

To provide a general overview of the changes in metabolites of algae before and after phycoremediation, a principal component analysis (PCA) was performed. The PCA results showed a clear distinction between the metabolic profiles of algae before and after phycoremediation, highlighting both similarities and differences at various levels. The hypothesis of this study was that different algal species exhibit distinct biochemical profiles before phycoremediation and that these profiles, together with elemental composition, undergo significant shifts following treatment. Multivariate statistical analyses, including PCA, AHC, and boxplot evaluations, were used to test this hypothesis because of their ability to capture complex variation, identify key discriminating traits, and validate clustering patterns across species.

Before phycoremediation (Figure 8), *Scenedesmus* sp. emerged as the most biochemically distinct species, primarily influenced by lipids and antioxidant activity (DPPH), while *Monoraphidium* sp. displayed the least variation. The clustering of traits such as lipids with DPPH and pigments with secondary metabolites indicated functional linkages among biochemical parameters, while boxplots highlighted broader variability in *Scenedesmus* sp. and *Klebsormidium* sp. compared to the relatively uniform *Monoraphidium* sp. These findings supported the hypothesis that species maintain distinct biochemical identities even under similar environmental conditions.

After phycoremediation (Figure 9), species separation became more pronounced, confirming that remediation altered algal biochemical composition. *Monoraphidium* sp. and *Nephrocytium* sp. aligned strongly with the first principal component and showed the highest variability, suggesting a greater biochemical responsiveness to remediation, whereas *Chloreococcum* sp. and *Klebsormidium* sp. remained relatively stable. These results demonstrate that phycoremediation does not affect all species equally but rather induces species‐specific shifts in biochemical profiles, thereby enhancing interspecific divergence.

In addition to biochemical traits, elemental composition analysis revealed further evidence of remediation effects (Figure 10). PCA and AHC clearly separated pre‐ and post‐treatment samples, with nitrogen (N%) and sulfur (S%) driving separation along one axis and carbon (C%) and hydrogen (H%) along another. Nitrogen emerged as the most variable and responsive element across treatments, while sulfur and hydrogen remained stable, and carbon showed intermediate variability. These elemental changes suggest that nitrogen metabolism, in particular, is highly influenced by phycoremediation, possibly linked to nutrient uptake and assimilation processes.

Taken together, these results confirm the hypothesis by showing that algae possess distinct biochemical and elemental signatures that are further reshaped by phycoremediation. The combination of PCA, AHC, and boxplot analyses provided complementary perspectives: score plots revealed species distribution, biplots identified trait contributions, clustering validated similarities, and boxplots highlighted variability. Collectively, Figures 8, 9, 10 demonstrate that phycoremediation enhances interspecific biochemical divergence and induces significant shifts in elemental stoichiometry, with nitrogen emerging as the key driver of post‐treatment differentiation.

### 4.2 Correlation Matrix

Metabolic analysis of algae after phycoremediation shows *Monoraphidium* sp., *Nephrocytium* sp., and *Scenedesmus* sp. have a positive correlation while showing a negative correlation with *Nephrocytium* sp. and *Klebsormidium* sp. *Chlorococcum* sp. and *Klebsormidium* sp. are positively correlated to each other and negatively correlated with the other three algae (Table 2A). The correlation matrix of CHNS shows nitrogen and sulfur show a positive correlation with each other. Carbon shows a positive correlation with hydrogen, and nitrogen and sulfur show a negative correlation with carbon and hydrogen (Table 2B).

**TABLE 2A:** Correlation matrix of Metabolite in algae before and after Phycoremediation.

Algal species	*Monoraphidium* sp.	*Scenedesmus* sp.	*Nephrocytium* sp.	*Chlorococcum* sp.	*Klebsormidium* sp.
*Monoraphidium* sp.	1	0.25862	0.51503	−0.65315	−0.0689
*Scenedesmus* sp.	0.25862	1	−0.25362	−0.19249	−0.03309
*Chlorococcum* sp.	0.51503	−0.25362	1	−0.86684	−0.42049
*Nephrocytium* sp.	−0.65315	−0.19249	−0.86684	1	0.5792
*Klebsormidium* sp.	−0.0689	−0.03309	−0.42049	0.5792	1

**TABLE 2B:** Correlation matrix of CHNS in algae before and after Phycoremediation.

CHNS	N%	C%	H%	S%
N%	1	−0.67612	−0.08656	0.76778
C%	−0.67612	1	0.48127	−0.50942
H%	−0.08656	0.48127	1	−0.1392
S%	0.76778	−0.50942	−0.1392	1

## 5 Conclusion

The present highlights the significant potential of algae as a tool for water pollution remediation due to their adaptability and biochemical responses to environmental stress. The analysis of five algal species exposed to Yamuna River water contaminated with various pollutants revealed notable biochemical changes. Decreases in chlorophyll a and b were observed across all species, while carotenoids, phenolic compounds, and flavonoids increased, particularly in *Chlorococcum* sp., suggesting adaptive metabolic responses. Additionally, antioxidant activity improved across all species, and lipid content varied, with some species showing increases while others exhibited reductions. CHNS analysis further revealed an increase in carbon content and a decrease in nitrogen and sulfur. PCA provided deeper insights into the correlations and metabolic shifts occurring due to the contaminants. These findings not only emphasize algae's resilience to stress but also their potential for sustainable applications, including biofuel production and natural antioxidants. This study contributes valuable knowledge on algae's dual role in environmental remediation and resource generation, offering pathways for sustainable water management and industrial applications.

## Conflicts of Interest

The authors declare no conflicts of interest.

## Data Availability

All data supporting the findings of this study are freely available. The five algal species (*Monoraphidium* sp., *Scenedesmus* sp., *Nephrocytium* sp., *Chlorococcum* sp., and *Klebsormidium* sp.) have been deposited in the Department of Botany, University of Delhi Microbial Collection Center.
